# Transactions of the Philosophical and Literary Society of Leeds

**Published:** 1838-01

**Authors:** 


Art. I.—*Transactions of the Philosophical and Literary Society of Leeds; consisting of Papers read before the Society.* Vol. I. Part 1. —*London*, 1837. 8vo. pp. 201.

We are glad to find that the Philosophical and Literary Society of Leeds is beginning to follow the example of some of the older institutions of a similar kind in this country, (for instance, that of the town of Manchester,) in publishing a selection of the papers read at the different meetings of the members. This is highly desirable in all institutions, the object of which is the dissemination of useful knowledge. We trust that other towns will follow the examples now set them by Leeds as well as Manchester; they will thus prove the means of recording much valuable information, which otherwise may be lost to the public, or at most be only partially disseminated through the neighbourhood of the local Society. A proof of the justice of this remark is offered by the volume before us, which contains several admirable papers, some of which were read as long ago as 1824-25. The nature of our Journal permits us to notice only those which are somewhat connected with our profession; but we may remark, in passing, that the others are not of inferior merit.

The papers which we feel ourselves more particularly called upon to notice are descriptive of the anatomy of three interesting genera of invertebrated animals. The first of these papers, on the Internal Anatomy of the *Limaces* found in the neighbourhood of Leeds, by Mr. Nunneley, is exceeding well written, and comprehends the complete anatomy of four species of Limax, accompanied by etchings of the parts described, which do equal honour to the anatomist and to the artist. The anatomy of the nervous system is beautifully executed, and, as we can state from our own observation, faithfully dissected and delineated; as are also the alimentary canal and the exterior of the animals themselves. We should, however, have been better pleased had the author been more minute in the descriptions he has given of the different structures, which we feel confident he could well have been.

The second paper, by Mr. Teale, is on the Anatomy of the *Actinia coriacea*, or Sea Anemone, a curious genus of animals, which has excited much interest among naturalists. The muscular system of this species, with the digestive and reproductive organs, are well described. It is with much satisfaction that we find Mr. Teale has observed the existence of cilia on the Tentacula, and has noticed also the direction of the currents they produce; although, as the author remarks, they had previously been noticed by Dr. Sharpey, and are since described by him in the article *Cilia*, in the Cyclopædia of Anatomy and Physiology. With regard to the much-disputed subject of the existence of a nervous system in the Actinia, Mr. Teale appears to have been as much unable to detect it as preceding anatomists; although a supposed nervous system in Actinia was long ago described by Spix. Mr. Teale has however observed, as also had M. Blainville, the existence of a grey pulpy cord, within the substance of the lip or mouth, which might probably be the nervous system; but he has been unable to satisfy himself that this is really the case, in consequence of its softness, which rendered him unable to trace it. Altogether, the paper has contributed much to our knowledge of this curious class of animals.

The next, and last, paper we are enabled to notice in this interesting volume is on the *Alcyonella stagnorum* of Lamark, a species of zoophyte found in the vicinity of Leeds, and now for the first time recorded as a British species. The general characters of this species, with its digestive and reproductive apparatus, are well described; and the paper is very creditable to its author as a naturalist.—On the whole, this first volume of the Literary and Philosophical Society of Leeds gives great promise of the future, and we hope that the example set by it will not be lost sight of by other Societies of a similar nature.

